# Risk stratification for small for gestational age for the Brazilian population: a secondary analysis of the Birth in Brazil study

**DOI:** 10.1038/s41598-020-71252-y

**Published:** 2020-09-07

**Authors:** Renato T. Souza, Matias C. Vieira, Ana Paula Esteves-Pereira, Rosa Maria Soares Madeira Domingues, Maria Elisabeth Lopes Moreira, Edson Vieira da Cunha Filho, Jane Sandall, Jose G. Cecatti, Maria do Carmo Leal, Marcos Augusto Bastos Dias, Dharmintra Pasupathy

**Affiliations:** 1 Department of Obstetrics and Gynaecology, School of Medical Sciences, University of Campinas (UNICAMP), 101 Alexander Fleming St, Cidade Universitaria, Campinas, SP Brazil; 2 Department of Women and Children’s Health, School of Life Course Sciences, Faculty of Life Sciences and Medicine, King’s College London, Women’s Health Academic Centre KHP, 10th Floor North Wing, St. Thomas’ Hospital, Westminster Bridge Road, London, SE1 7EH UK; 3 Department of Obstetrics and Gynaecology, São Lucas Hospital, School of Medicine, Pontifical Catholic University of Rio Grande Do Sul, 6690 Ipiranga Avenue, Porto Alegre, RS Brazil; 4 National School of Public Health, Oswaldo Cruz Foundation/FIOCRUZ, 1480 Leopoldo Bulhoes St, Manguinhos, Rio de Janeiro, RJ Brazil; 5 National Institute of Infectious Diseases, Oswaldo Cruz Foundation/FIOCRUZ, 4365 Brasil Avenue, Manguinhos, Rio de Janeiro, RJ Brazil; 6 Department of Neonatology, Fernandes Figueira Institute, Oswaldo Cruz Foundation/FIOCRUZ, 716 Rui Barbosa st, Flamengo, Rio de Janeiro, Brazil; 7 Fernandes Figueira Institute, Oswaldo Cruz Foundation/FIOCRUZ, 716 Rui Barbosa st, Flamengo, Rio de Janeiro, Brazil; 8 Discipline of Obstetrics, Gynaecology and Neonatology, Westmead Clinical School, University of Sydney, Sydney, NSW 2145 Australia

**Keywords:** Risk factors, Medical research, Epidemiology, Population screening

## Abstract

Risk-stratification screening for SGA has been proposed in high-income countries to prevent perinatal morbidity and mortality. There is paucity of data from middle-income settings. The aim of this study is to explore risk factors for SGA in Brazil and assess potential for risk stratification. This population-based study is a secondary analysis of Birth in Brazil study, conducted in 266 maternity units between 2011 and 2012. Univariate and multivariate logistic regressions were performed, and population attributable fraction estimated for early and all pregnancy factors. We calculated absolute risk, odds ratio, and population prevalence of single or combined factors stratified by parity. Factors associated with SGA were maternal lupus (OR_adj_ 4.36, 95% CI [2.32–8.18]), hypertensive disorders in pregnancy (OR_adj_ 2.72, 95% CI [2.28–3.24]), weight gain < 5 kg (OR_adj_ 2.37, 95% CI [1.99–2.83]), smoking at late pregnancy (OR_adj_ 2.04, 95% CI [1.60–2.59]), previous low birthweight (OR_adj_ 2.22, 95% CI [1.79–2.75]), nulliparity (OR_adj_ 1.81, 95% CI [1.60–2.05]), underweight (OR_adj_ 1.61, 95% CI [1.36–1.92]) and socioeconomic status (SES) < 5th centile (OR_adj_ 1.23, 95% CI [1.05–1.45]). Having two or more risk factors (prevalence of 4.4% and 8.0%) was associated with a 2 and fourfold increase in the risk for SGA in nulliparous and multiparous, respectively. Early and all pregnancy risk factors allow development of risk-stratification for SGA. Implementation of risk stratification coupled with specific strategies for reduction of risk and increased surveillance has the potential to contribute to the reduction of stillbirth in Brazil through increased detection of SGA, appropriate management and timely delivery.

## Introduction

Small for gestational age (SGA) is usually defined as a newborn with birth weight below the 10th centile for gestational age by either population, customized or other international birthweight centiles charts^1–3^. Some SGA infants are constitutionally small whilst some are due to fetal growth restriction (FGR). All SGA infants are at increased risk of perinatal morbidity and mortality^4^; however, short- and long-term adverse outcomes are more strongly associated with FGR^3,5,6^. Antenatal detection of SGA allows for appropriate follow up and timely delivery and is associated with a reduction in adverse perinatal outcomes including stillbirth^4,5^.

High-income countries such as United Kingdom (UK), Canada, United States, France, Ireland, and New Zealand have adopted national guidelines for screening and managing SGA cases that are based on risk-stratified algorithms^1^. Current clinical management is often focused on early identification of women at risk of SGA to ensure a stratified and appropriate level of surveillance and care^2,3^. Saving Babies’ Lives, a recent initiative to reduce stillbirths in England^7^, has introduced a care bundle with multiple components; one of which is based on risk-assessment algorithms and surveillance for SGA and it has demonstrated 58% improvement in antenatal detection of SGA. Evaluation of the impact of this care bundle has also reported a 20% decrease in the rate of stillbirth^8,9^.

Brazil has approximately 3 million live births (LB) annually and in 2016 the stillbirth rate was 10.4/1,000 live births, ranging from 8.2 to 14.7 in the different states^10,11^. National guidelines recommend symphysis fundal height measurement as the primary method for screening for SGA for both low and high-risk pregnancies^12,13^. Only one ultrasound is currently recommended as routine during antenatal care for low-risk women with the aim of accurate estimation of gestational age^12^. Additional scans for fetal growth assessment are recommended only on an individual base if a clinical suspicion arises^12^. Geographical and social inequalities of health care coverage and limited resources of the Unified Public Health System (SUS) remain a great challenge in Brazil^14^. A risk stratification-based screening strategy may be an effective approach to balance the provision of ultrasound to women who would benefit most from increased surveillance to better detect SGA fetuses and therefore potentially improving perinatal outcomes.

While risk factors for SGA have widely been studied, few reports are from and middle-income countries. Understanding of size of effect, as well as prevalence and population attributable fraction for each risk factor, is necessary to estimate potential effect and provision required for implementing risk stratification for SGA in pregnancy. Therefore, the aim of this study is to explore risk factors for SGA in Brazil and assess the potential for risk stratification.

## Methods

This is a secondary analysis of the Birth in Brazil study, a nationwide hospital based survey conducted in 266 maternity units in Brazil from February 2011 to October 2012^15^. The cohort included women who had live births (regardless birthweight or gestational age) and stillbirths (birthweight ≥ 500 g and/or gestational age ≥ 22 weeks) in hospitals with more than 500 deliveries/year. The study excluded women who had a history of severe mental health disorder, who were homeless, did not understand Portuguese, with speech or hearing impairment, and were sectioned by court order. Information on the complex sampling method performed in the Birth in Brazil is published in detail else where^15^. In brief, a two-stage sampling of hospitals and women followed rigorous procedures to assure representativeness of the Brazilian population according to the five geographical regions, type of municipality (capital or not capital) and type of governance (public, private and mixed), resulting in a sample size of 90 women per hospital from 191 municipalities. This provided a cohort of 23,940 women and 24,200 live births.

Face-to-face interviews with women within 24 h following delivery were undertaken after recruitment and review of antenatal and postnatal medical records were carried out. Research assistants completed electronic questionnaires to obtain information on socioeconomic status, maternal anthropometrics and lifestyle, clinical history, obstetric history, and data related to the antenatal, intrapartum and postnatal period. Gestational age at delivery was estimated based on different sources of information with the aim of maximizing ascertainment of gestation in days, rather than complete weeks. The following hierarchical approach was used: (1) first ultrasound (US) (at any gestational age), (2) LMP recorded on maternal medical records, (3) LMP reported by the woman in the interview, (4) gestation age in number of complete weeks recorded on admission, (5) gestation age in number of complete weeks reported during interview.

All participating women in the Birth in Brazil study gave informed consent and several procedures were held to ensure anonymity and confidentiality of participant’s identity. The Ethics Committee for Research at Public Health National School, Oswaldo Cruz Foundation (ENSP/ Fiocruz), approved the Birth in Brazil study (letter of approval number 92/2010). No additional ethical approval was necessary for this secondary analysis. The study was developed in compliance with the Declaration of Helsinki guidelines (Finland, 1964) for studies in humans.

In this analysis the study population comprised singleton pregnancies without fetal anomalies, with birth between 24 and 43 weeks of gestation, and with available information on birthweight, sex and gestational age.

### Outcomes and exposures

The primary outcome for this study was small for gestational age (SGA) newborns, which was defined based on a birthweight below the 10th population centile (< p10) adjusted for gestational age and sex. Population centiles were internally developed using the LMS (Lambda-Mu-Sigma) method using data from women without pregnancy complications such as hypertensive disorders of pregnancy, diabetes and smoking. Secondary outcomes included information on pregnancy (preterm birth rate, mode of delivery, perineal tears and maternal near miss as defined by the World Health Organization^16^), and on perinatal status (birth weight, Apgar score, NICU admission, severe neonatal morbidity, neonatal near miss^17^, stillbirth and perinatal mortality). Severe neonatal morbidity was defined as having any of the following: mechanical ventilation, seizures, pulmonary hypertension, requirement for chest compression at birth, sepsis, birth asphyxia.

Exposures of interest were selected on the basis of known and available potential maternal and pregnancy characteristics associated with SGA. These include maternal age (< 35, 35–39, ≥ 40 years), ethnicity, body mass index (BMI), model of care (privately or publicly funded), partner status (living with or not living with), parity, low socioeconomic status (< 5th centile in this study population), previous low birth weight infant, previous pregnancy loss (mainly but not exclusively stillbirth), chronic hypertension, chronic kidney dysfunction (CKD), systemic lupus erythematosus, pre-existing diabetes, smoking in early pregnancy, smoking late in pregnancy, maternal weight gain (< 5 kg, 5–20 kg, > 20 kg), hypertension in pregnancy (includes preeclampsia and pregnancy induced hypertension), and gestational diabetes (as recorded in medical record). Gestational weight gain was calculated by the difference between the first and last available maternal weights as recorded in the antenatal chart. The socioeconomic status score was calculated according to the ABEPEMI, the Brazilian Association of Market Survey Institutes; the score were then divided into five social classes (A, B, C, D and E), according to the following cut points: 35–46 (A), 23–34 (B), 22–14 (C), 8–13 (D) and 0–7 (E). The first maternal weight was based on the self-reported weight just before pregnancy and on the last weight measured in pregnancy. Maternal BMI was categorized into underweight (< 18.5 kg/m^2^), normal weight (18.5–24.9 kg/m^2^), overweight (25.0–29.9 kg/m^2^), and obesity (≥ 30.0 kg/m^2^), calculated using the self-reported weight just before pregnancy and the self-reported height. Total weight gain was calculated based on the difference between the self-reported weight just before pregnancy and the last weight measured in pregnancy.

### Statistical analysis

All women with data on birthweight and gestational at delivery were included in the analysis. Descriptive statistics were provided using mean (± SD) and frequency (percentages). SGA infants and non-SGA infants (including large for gestational age infants) were compared using student-t test and chi-squared test, as appropriate. Univariable and multivariable logistic regression were used to explore the size of effect of risk factors. Reference categories used were: maternal age 20–34 years, Estimation of prevalence of each exposure and population attributable fraction (PAF) of risk factors were performed. Multivariable analysis was adjusted for both early pregnancy factors (exposures detected at the beginning of pregnancy) and all factors (separate models reported). Risk factors were combined to assess the potential for risk stratification in nulliparous and multiparous women separately. Risk stratification for early and late pregnancy was reported.

All analyses accounted for the complex sampling design. For logistic regression models, we did not assume normal distribution or linear association. A significance level of 5% was considered for all analyses. The statistical package used was STATA software, version 15.0 (StataCorp, Texas, USA).

### Role of the funding source

The funders played no role in the study design, data collection/analysis, interpretation of data, decision to publish or manuscript preparation.

## Results

**Figure 1:**
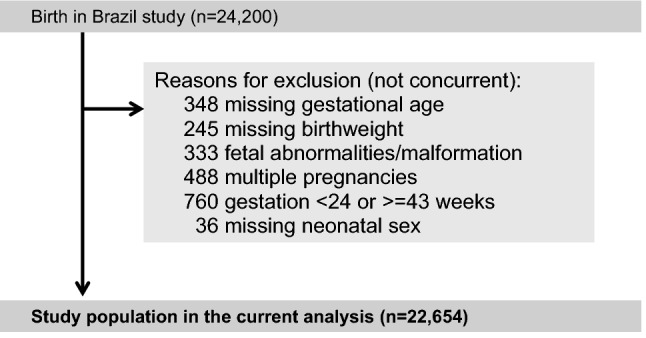
Flowchart.

Amongst 24,200 births from the Birth in Brazil study, 22,654 women were included in the current analysis (Fig. 1). The rate of SGA was 11.1% (n = 2,481). Maternal sociodemographic, clinical characteristics and perinatal outcomes are reported in Table 1. Women with SGA infants were more likely nulliparous, underweight, of lower socioeconomic status, with a previous low birthweight infant, and received care in the public sector. These women also had higher rates of adverse maternal and perinatal outcomes, such as preterm birth (< 37 weeks and < 34 weeks), maternal near miss, NICU admission, severe neonatal morbidity and neonatal near miss, stillbirth and perinatal mortality. Perinatal mortality and stillbirth were 2–3 times higher in SGA infants.

**Table 1:** Demographic characteristics, maternal and perinatal outcomes.

Characteristics	SGA		Non-SGA		*p* value
	(n = 2,481)		(n = 20,173)		
	Mean (SD) or n (%)		Mean (SD) or n (%)		
Age (years)^a^	25.4	(6.7)	25.7	(6.4)	0.56
**Ethnicity/skin colour**^a^					
White	807	(31.1)	7,361	(34.1)	**0.01**
Black	216	(9.4)	1,552	(8.6)	
Brown	1,426	(58.3)	10,944	(55.7)	
Asian	17	(0.6)	235	(1.2)	
Other	14	(0.5)	77	(0.4)	
Multiparous	1,136	(46)	10,898	(53.8)	** < 0.001**
Previous caesarean section^b^	420	(36.3)	4,696	(40.6)	**0.03**
previous low birthweight	269	(24.1)	1,277	(11.8)	** < 0.001**
previous pregnancy loss	70	(6.3)	435	(4.2)	**0.01**
Height (cm)^c^	159.1	(7.2)	161	(7.1)	** < 0.001**
Weight (kg)^d^	56.7	(11.8)	60.4	(12.4)	** < 0.001**
**BMI category**^e^					
Underweight	227	(12.6)	1,236	(8.6)	** < 0.001**
Normal weight	1,152	(65.3)	9,775	(61.6)	
Overweight	293	(15.5)	3,306	(20.8)	
Obesity	114	(6.6)	1,408	(9)	
Gestational weight gain (kg)^f^	11.0	(6.6)	12.9	(6.4)	** < 0.001**
Socioeconomic status* (score)^g^	17.3	(6.6)	18	(6.8)	** < 0.001**
A + B	583	(20.3)	5,775	(25)	** < 0.001**
C	1,202	(52.7)	9,834	(51.8)	
D + E	674	(26.9)	4,375	(23.2)	
Single mother^h^	493	(21.1)	3,401	(18.3)	**0.01**
Private care	521	(15.4)	5,519	(20.7)	** < 0.001**
**Pregnancy comorbidities**^i^					
Chronic hypertension	65	(2.8)	454	(2.5)	0.26
Preeclampsia/Eclampsia	418	(18.1)	1,878	(10.1)	** < 0.001**
Pre-existing diabetes	12	(0.5)	194	(1)	0.09
Gestational diabetes	127	(5.4)	1,510	(7.8)	** < 0.001**
**Pregnancy outcomes**					
Gestation at delivery	38.2	(2.9)	38.9	(2.1)	** < 0.001**
Very preterm (< 34 weeks)	179	(10)	458	(2.8)	
Preterm (< 37 weeks)	476	(21.7)	2,084	(11.5)	
At 37 weeks	282	(10.7)	2,108	(10.2)	
At 38 weeks	478	(17.9)	4,889	(22.5)	
At 39 weeks	567	(22.3)	5,396	(26.1)	
At 40 weeks	411	(16.8)	3,668	(19.5)	
At 41 weeks	178	(7.2)	1,585	(8.1)	
At 42 weeks	89	(3.4)	443	(2)	
**Mode of delivery**					
Spontaneous vaginal	1,175	(49.9)	8,474	(46.8)	** < 0.001**
Forceps/ventous	15	(0.5)	276	(1.6)	
Caesarean section (any)	1,291	(49.6)	11,423	(51.6)	
3rd /4th degree tear^j^	1	(0.1)	30	(0.3)	0.21
Maternal Near Miss^i^	42	(2)	139	(0.9)	** < 0.001**
**Perinatal outcomes**					
Birthweight	2,361.3	(469.6)	3,276	(456.9)	** < 0.001**
Apgar below 7 at 5 min^k^	34	(1.4)	126	(0.7)	**0.02**
NICU admission^l^	299	(14.1)	658	(3.8)	** < 0.001**
Severe neonatal morbidity^m^	112	(6.5)	309	(1.8)	** < 0.001**
Neonatal Near Miss^n^	553	(24.4)	1,546	(8.4)	** < 0.001**
Stillbirth	45	(2.1)	45	(0.2)	** < 0.001**
Perinatal mortality	78	(3.9)	98	(0.5)	** < 0.001**

BMI: body mass index; NICU, neonatal intensive care unit; SD, standard deviation; SGA, small for gestational age. *p* values in bold are statistically significant.

Missing information for ^a^5; ^b^62; ^c^4,674; ^d^1,219; ^e^5,143; ^f^1,760; ^g^211; ^h^12; ^i^23; ^j^13,630; ^k^882; ^l^108; ^m^879; ^n^90 cases.

*Socioeconomic status was reported as both continuous (score) and categorical (social class).

In Table 2, the population prevalence of potential risk factors of SGA, univariable and multivariable and population attributable fraction for SGA are reported. The most prevalent factors include nulliparity (47.1%), age < 20 (19.2%), single motherhood (18.6%), hypertensive disorder in pregnancy (11.0%), smoking in early pregnancy (9.3%) and underweight (9.0%). Factors independently associated with SGA infants were maternal lupus (OR_adj_ 4.36, 95% CI [2.32–8.18]; PAF 0.3%), hypertensive disorders in pregnancy (OR_adj_ 2.72, 95% CI [2.28–3.24]; PAF 11.2%), weight gain < 5 kg (OR_adj_ 2.37, 95% CI [1.99–2.83]; PAF 6.0%), smoking at late pregnancy (OR_adj_ 2.04, 95% CI [1.60–2.59]; PAF 4.8%), previous low birthweight (OR_adj_ 2.22, 95% CI [1.79–2.75]; PAF 4.7%), nulliparity (OR_adj_ 1.81, 95% CI [1.60–2.05]; PAF 24.1%), underweight (OR_adj_ 1.61, 95% CI [1.36–1.92]; PAF 4.3%) and socioeconomic status (SES) < 5th centile (OR_adj_ 1.23, 95% CI [1.05–1.45]; PAF 0.7%).

**Table 2:** Potential risk factors for SGA—Univariate and multivariate (early and all factors) analyses, population prevalence and population attributable fractions.

Exposures	Population prevalence (%)	Univariate analysis	Multivariable—early pregnancy*	Multivariable—all factors**	
		OR (95% CI)	OR_adj_ (95% CI)	OR_adj_ (95% CI)	PAF (%)
Age > 40 years	1.9	1.30 (0.95–1.77)	1.34 (0.93–1.93)	1.27 (0.83–1.92)	0.4
Age 35–39.9 years	8.5	1.13 (0.92–1.39)	1.23 (0.99–1.52)	1.15 (0.95–1.41)	1.1
Age < 20 years	19.2	1.26 (1.10–1.44)	0.99 (0.85–1.14)	1.01 (0.86–1.18)	0.2
Nulliparous	47.1	1.37 (1.23–1.53)	1.89 (1.67–2.14)	1.81 (1.60–2.05)	24.1
Underweight	9.0	1.38 (1.15–1.67)	1.40 (1.17–1.67)	1.61 (1.36–1.92)	4.3
Obesity	8.8	0.69 (0.58–0.84)	0.77 (0.63–0.95)	0.50 (0.39–0.65)	−5.2
Weight gain < 5 kg	7.4	1.86 (1.42–2.43)		2.37 (1.99–2.83)	6.0
Weight gain > 20 kg	9.8	0.69 (0.55–0.88)		0.55 (0.43–0.72)	−4.4
Single mother	18.6	1.19 (1.03–1.38)	1.04 (0.90–1.20)	0.98 (0.83–1.15)	−0.3
SES < 5th centile	5.5	1.46 (1.21–1.76)	1.27 (1.08–1.49)	1.23 (1.05–1.45)	0.7
Previous stillbirth	2.4	1.28 (0.99–1.65)	1.12 (0.69–1.80)	1.15 (0.71–1.87)	0.2
Previous low birthweight	6.9	1.84 (1.50–2.25)	2.46 (1.95–3.10)	2.22 (1.79–2.75)	4.7
Smoking 1st trimester	9.3	1.90 (1.62–2.23)	1.78 (1.45–2.19)		
Smoking until 3rd trimester	7.2	2.04 (1.80–2.31)		2.04 (1.60–2.59)	4.8
Lupus	0.1	4.41 (1.61–12.1)	4.08 (2.12–7.83)	4.36 (2.32–8.18)	0.3
Chronic kidney disease	0.2	2.37 (0.82–6.90)	2.30 (0.59–8.98)	2.15 (0.37–12.63)	0.2
Chronic hypertension	2.5	1.14 (0.83–1.57)	1.12 (0.75–1.66)	0.76 (0.50–1.14)	−0.7
Hypertensive disorder in pregnancy	11.0	1.97 (1.66–2.33)		2.72 (2.28–3.24)	11.2
Previous diabetes	1.0	0.51 (0.30–0.87)	0.63 (0.41–0.99)	0.60 (0.38–0.96)	−0.4
Gestational diabetes	7.6	0.67 (0.51–0.88)		0.70 (0.53–0.92)	−2.2

*SES* socioeconomic status, *OR* odds ratio, *CI* confidence interval, *PAF* population attributable fraction, *SGA* small for gestational age.

*Multivariable analysis adjusted for all early pregnancy factors.

**Multivariable analysis adjusted for all pregnancy factors, except smoking 1st trimester which was replaced by smoking until 3rd trimester.

**Figure 2:**
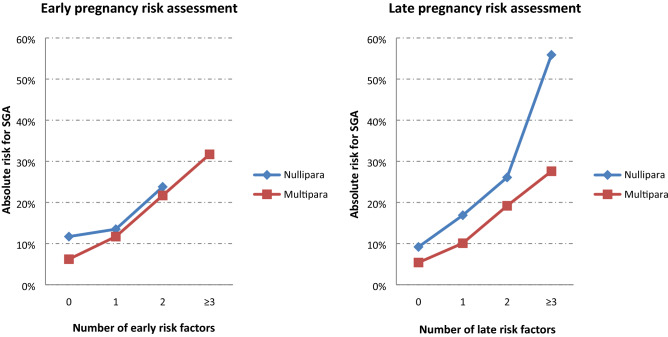
Absolute risks for SGA according to the number of risk factors: early and late pregnancy risk assessments.

In Table 3, the prevalence, absolute risk and odds ratio for SGA of a combination of increasing number of risk factors identified in multivariable analysis are reported for nulliparous and multiparous women. Results are also reported stratified by early and late pregnancy. In nulliparous women, in early pregnancy, those without additional risk factors had SGA rates similar to the background population. The odds of SGA increased more than two-fold when two or more risk factors were present. In late pregnancy of nulliparous women, when risk assessment included both factors present in early pregnancy and those that developed during pregnancy, the presence of one or more risk factors increased the odds of SGA greater than two-fold. There was a dose-dependent relationship between the number of risk factors and the odds of SGA. A similar association was observed in multiparous women though the baseline rate of SGA was lower in those without risk factors and even in the presence of risk factors the rate of SGA was lower than among nulliparous women (Fig. 2).

**Table 3:** Risk of SGA, population prevalence and composite neonatal outcomes according to a combination of risk factors.

	Prevalence (%)	Risk of SGA (%)	OR	(95% CI)
**Nulliparous**				
**Early pregnancy assessment**^**a**^				
No additional risk factor	81.9	11.7	ref	
1 additional risk factor	16.7	13.5	1.18	(0.95–1.46)
2 additional risk factors	1.4	23.8	2.35	(1.46–3.78)
3 or more additional risk factors	0.0	100	–	–
**Late pregnancy assessment**^**b**^				
no additional risk factor	67.9	9.2	ref	
1 additional risk factor	28.6	16.9	2.01	(1.63–2.48)
2 additional risk factors	3.4	26.1	3.48	(2.50–4.83)
3 or more additional risk factors	0.1	55.9	12.52	(3.02–51.85)
**Multiparous**				
**Early pregnancy assessment**^**a**^				
no risk factor	71.0	6.2	ref	
1 risk factor	24.2	11.7	1.99	(1.63–2.44)
2 risk factors	4.4	21.7	4.16	(2.83–6.11)
3 or more risk factors	0.4	31.7	6.98	(4.14–11.77)
**Late pregnancy assessment**^**b**^				
no risk factor	60.1	5.4	ref	
1 risk factor	30.9	10.1	1.97	(1.69–2.30)
2 risk factors	8.0	19.2	4.20	(3.22–5.49)
3 or more risk factors	1.0	27.6	6.71	(3.91–11.52)

*CI* confidence interval, *OR* odds ratio, *Ref* reference, *SGA* small for gestational age.

^a^Early pregnancy factors: underweight, lupus, smoking and previous low birthweight; ^b^All pregnancy factors: underweight, lupus, smoking, previous low birthweight, weight gain < 5 kg and preeclampsia.

## Discussion

We evaluated risk factors for SGA in a representative sample of the Brazilian population, according to the available relevant maternal and pregnancy conditions potentially associated to SGA. A risk stratification model was developed including early and late factors such as maternal lupus, hypertensive disorders of pregnancy, smoking during pregnancy, low gestational weight gain (< 5 kg), previous low birthweight infant, SES < 5th centile, underweight, and nulliparity. Nulliparity was independently associated with SGA. In the stratified analysis by parity the rate of SGA amongst women with no early or late risk factors was 9.2% in nulliparous women and 5.4% in multiparous women. The absolute risk for SGA increased with increasing number of risk factors although the population prevalence of women with multiple risk factors decreased. Similar associations were observed when the analysis was confined to early pregnancy risk assessment only.

The primary goal of risk stratification for SGA is to inform clinical surveillance and improve antenatal detection of these fetuses. The latter allows for appropriate management and timely delivery, with the aim of avoiding stillbirth. This approach is implemented in England, through the Saving Babies Lives care bundle^7^. According to an independent evaluation of its introduction in 17 trusts, there was an increase of 25.7% in the number of ultrasounds after two years, resulting in increased antenatal detection of SGA and decreased stillbirth rate^8^. Although the results cannot yet be fully attributed to the bundle of actions, these results are encouraging as they exceeded the national rate of decline in the period^8^. The implementation of such strategy in middle-income countries, however, still requires further investigation on the performance of the detection of SGA using risk stratification-based screening (e.g. serial fundal height for low-risk women and serial/timely provision of ultrasounds for higher-risk women). Conversely and alternative strategy such as in France universal third-trimester scan is recommended^18^. Providing additional universal ultrasound scans is unlikely to be feasible in middle income countries. Our results provide an opportunity for an alternative screening strategy that is relevant for the Brazilian setting and other similar settings. Women with 2 or more risk factors (irrespective of parity status) had a risk of SGA greater than 20%, approximately double the background risk. This group would represent 4% and 8% of nulliparous and multiparous women, respectively. Providing scans for these groups (approximately 6% of the population) would more likely be acceptable in settings such as Brazil. There is a trade-off between the number of factors required to define high risk and the service provision needed for increased surveillance with ultrasound scans.

Another advantage of an early pregnancy risk stratification tool is the potential for prevention. Some of the independent risk factors for SGA in this study were potentially modifiable, such as maternal anthropometric parameters (underweight and weight gain < 5 kg) and smoking. Monitoring maternal weight gain as a risk factor during pregnancy is feasible in most settings, not time-consuming and reproducible at population level. A systematic review with meta-analysis including 11 observational studies showed that weight gain below the recommended level by guidelines was associated with a 53% greater risk for SGA (95% CI [1.44–1.64])^19^. A greater increase in the risk was observed for underweight women (OR 1.89, 95% CI [1.67–2.14])^19^. Smoking in pregnancy was also another modifiable risk factor in our study. Women who ceased smoking at the beginning of pregnancy showed to have the same risk for SGA as women who had never smoked^20^. Addressing smoking cessation and nutritional needs would not only affect the risk for SGA but for many other maternal and perinatal complications. Indeed, Brazilian guidelines already recognize smoking cessation and weight management during pregnancy and pathways of care have already been recommended^12,13^. Risk stratification for SGA, such as proposed by the results of this study, would ensure these women are identified for opportune and appropriate referral. In addition to addressing the modifiable risk factors above, there is potential for preventing SGA through interventions directed to high-risk women. Aspirin for preventing SGA infants is not one of the strategies currently recommended by the Brazilian guidelines^12^. The use of aspirin for women at higher risk for preeclampsia or SGA for instance seems to reduce the risk for SGA and has been recommended by international guidelines in high-income countries^1,21–23^. The ASPRE study, a randomized controlled trial that included 1,620 women at high-risk for preeclampsia, demonstrated that aspirin reduced the incidence of SGA infants born at any gestation age and < 37 weeks by 24% (OR 0.768, 95% CI [0.646–0.911]) and 40% (OR 0.607, 95% CI [0.415–0.889]), respectively^22^. The screening of women at higher risk for PE in both studies was based on maternal history, pregnancy characteristics, ultrasound and biochemical factors. In addition, the major contribution of aspirin was for those women who developed PE, although it was not limited to them. Therefore, the benefit of the use of aspirin to prevent SGA remains unclear, especially in middle-income countries such as Brazil. In practice, the implementation of screening tools can be complex due to its translatability. As an example, preeclampsia screening has not yet been introduced in many high income countries including the UK. Simpler screening tools are more translatable and can be useful, such as venous thrombotic embolism score^24^. Before moving to a complex model we are exploring more simple risk stratification tools. This information may not be as accurate as a complex modelling, but it may provide useful information for clinical use where there is no current risk stratification model, as currently in Brazil.

We did not establish what a proper gestational period for late pregnancy assessment was. Despite the uncertainty of what this period may represent, our findings supports the need for continuous monitoring during pregnancy as some of the conditions may develop in different moment of pregnancy. Some of the factors, such as the development of preeclampsia and the abnormal weight gain pregnancy, may only be present at the end of pregnancy. This would be useful to inform how dynamic the risk assessment may be in pregnancy, indicating that risk may change according to the development of abnormal conditions in late pregnancy and that the women would, now, require increased surveillance regarding fetal growth.

The main strength of this analysis is the nation-wide characteristic of Birth in Brazil and a study population representative of the Brazilian population with standardized procedures for sampling, data collection and data registering. The nature of the data collection, which included an interview with women with potential recall bias, may have limited our ability to identify some risk factors. For example, previous stillbirth may have an overlap with any pregnancy loss, given this information was mainly obtained from women interview after delivery rather than from medical records. Although data about chronic kidney disease, chronic hypertension and previous stillbirth were addressed, these conditions were not associated with SGA in our study. The low prevalence may have limited the power for identifying moderate sizes of effect for these conditions, particularly for chronic kidney disease and previous stillbirth. The statistical power of Birth in Brazil sample size calculation did not account for such conditions that are less common than the primary outcomes. Although the prevalence of chronic hypertension is in agreement with the literature^25^, it is not possible to exclude that there was an overlap with other hypertensive disorders of pregnancy. In addition, we do not address whether hypertension was considered controlled or not. We’ve noted SGA infants delivered earlier. Although this was possibly related to antenatal detection and timely delivery, it is also possible this represent the association of preterm birth with SGA. We acknowledge the fact that the application of a new centile chart than the one used clinically may also have influenced, although this is less likely.

In conclusion, early pregnancy risk stratification for SGA is considered a potentially effective strategy in high-income countries for improving the detection of SGA and consequentially reducing the risk of stillbirth and perinatal morbidity and mortality. One of the issues that limit the translation of these strategies to middle-income countries is the limited resources for increased surveillance. Understanding the epidemiology of SGA in these populations is required for estimating appropriateness, impact and resources needed to implement such strategy in different settings. Our results suggest risk stratification for SGA that is relevant to the Brazilian setting is achievable. Implementation of risk stratification coupled with specific strategies for reduction of risk and increased surveillance has the potential to contribute to reduction of stillbirth in Brazil through increased detection of SGA, appropriate management and timely delivery.

### Ethical approval

The Birth in Brazil study was approved by the Ethics Committee for Research at Public Health National School, Oswaldo Cruz Foundation (ENSP/Fiocruz)—number 92/2010, and in each participating center when appropriate.

## Data Availability

Birth in Brazil study database is not available in data repositories, but any data required can be provided by the Birth in Brazil team after appropriate approval.
